# Clinical predictors of drug-resistant epilepsy in children

**DOI:** 10.3906/sag-2010-27

**Published:** 2021-06-28

**Authors:** Pakize KARAOĞLU, Uluç YİŞ, Ayşe İpek POLAT, Müge AYANOĞLU, Semra HIZ

**Affiliations:** 1 Pediatric Neurology Clinic, University of Health Sciences Dr. Behçet Uz Child Disease and Pediatric Surgery Training and Research Hospital, İzmir Turkey; 2 Division of Pediatric Neurology, Department of Pediatrics, Faculty of Medicine, Dokuz Eylül University, İzmir Turkey

**Keywords:** Childhood, drug-resistant epilepsy, refractory seizures

## Abstract

**Background/aim:**

In up to 20% of epilepsy patients, seizures may not be controlled despite the use of antiepileptic drugs, either alone or in combination. These individuals are considered to have drug-resistant epilepsy. Drug-resistant epilepsy is usually associated with intellectual disability, psychiatric comorbidity, physical injury, sudden unexpected death, and low quality of life. Early detection and prediction of drug-resistant epilepsy are essential in determining the patient’s most appropriate treatment option. This retrospective study aimed to determine the clinical, electroencephalographic, and radiological factors associated with medically intractable childhood seizures.

**Materials and methods:**

Data regarding 177 patients diagnosed with drug-resistant epilepsy were compared with 281 patients with drug-responsive epilepsy.

**Results:**

Univariate analysis showed that age at seizure onset, having mixed seizure types, history of status epilepticus, history of neonatal seizures, history of both having febrile and afebrile seizures, daily seizures at the onset, abnormality on the first electroencephalogram, generalized epileptic abnormality on electroencephalogram, abnormal neurodevelopmental status, abnormal neuroimaging, and having symptomatic etiology were significant risk factors for the development of drug-resistant epilepsy (p < 0.05). In multivariable analysis, having mixed seizure types, history of status epilepticus, having multiple seizures in a day, intellectual disability, symptomatic etiology, and neuroimaging abnormality remained significant predictors for developing drug-resistant epilepsy.

**Conclusions:**

In the course of childhood epilepsy, some clinical features may predict the outcome. Early identification of patients with high risk for drug-resistant epilepsy will help plan the appropriate treatment option. Further prospective studies should confirm these findings.

## 1. Introduction

In most patients with epilepsy, seizures respond well to antiepileptic medication. Although antiepileptic drugs (AEDs) are used either alone or in combination, epileptic seizures cannot be controlled in 10%–20% of patients [1,2]. Although drug-resistant epilepsy patients constitute a minor part of the patients with epilepsy, they suffer from the significant psychosocial and economic burden of the disease and require considerable time and effort from the physician. Drug-resistant epilepsy has generally been associated with intellectual disability, psychiatric comorbidity, physical damage, sudden unexpected death, and low quality of life [3]. Children with refractory epileptic seizures use multiple antiepileptic drugs for long term, which may negatively affect their cognitive and physical development [3]. Early prediction of drug-resistant epilepsy may enable patients to be evaluated earlier in terms of alternative treatments such as ketogenic diet, epilepsy surgery, or vagal nerve stimulation. Providing patients with appropriate treatment in a timely manner may prevent the development of comorbidities associated with drug-resistant epilepsy [1,3].

This study aimed to determine the clinical, electroencephalographic, and radiological factors associated with intractable childhood seizures.

## 2. Materials and methods

This retrospective study was carried out at Dokuz Eylül University Faculty of Medicine, Department of Pediatric Neurology. The institutional ethics committee approved the study. Families of all participants gave informed consent before the study. The study included patients with epilepsy who were followed up for at least 2 years. Clinical data were obtained from patient’s medical records. Patient’s sex, age, age at onset of seizures, seizure type at the onset, initial seizure frequency, multiple seizures per day, etiology, previous history of status epilepticus, history of febrile and neonatal seizures, family history, mental and motor development, electroencephalogram abnormalities, brain magnetic resonance imaging (MRI) findings, specific epileptic syndromes, and behavioral problems were noted.

Epilepsy was defined as two or more unprovoked seizures. Drug-resistant and drug-responsive epilepsy were determined according to the International League against Epilepsy (ILAE) definition. Drug-resistant epilepsy was defined as the failure of adequate trials of two tolerated and appropriately chosen and used AED schedules (whether as monotherapy or in combination) to achieve sustained seizure freedom. Drug-responsive epilepsy was defined as epilepsy in which the patient receiving the current AED regimen has been seizure-free for a minimum of three times the longest preintervention interseizure interval or 12 months, whichever is longer [4]. Records of the patients with drug-resistant epilepsy and drug-responsive epilepsy were reviewed to identify the variables associated with seizure intractability.

Data were processed and analyzed using SPSS software. Descriptive statistics, the Pearson chi-squared test, likelihood ratios, continuity corrections, and the Fisher exact test were used to analyze data where applicable. The level of significance was set at p < 0.05. Finally, all variables demonstrating a significant association with outcomes were entered into a logistic regression model to determine independent predictors of intractable epilepsy. 

## 3. Results

There were 458 epileptic children (210 female and 248 male) with a mean age of 7.73 ± 4.72 years. The patients with at least two years of the follow-up period were included in the study. The median follow-up time of the patients was 46 months (range: 28–126 months). One hundred seventy-seven of them had drug-resistant epilepsy, and 281 had drug-responsive epilepsy. The high number of patients with drug-resistant epilepsy can be explained by the fact that our center is a tertiary pediatric neurology center where resistant epilepsy cases are referred. 

Sex and family history of seizures were not significantly different between the two groups. There was no statistically significant difference between drug-resistant epilepsy and drug-responsive epilepsy groups in terms of focal, generalized, and unknown onset seizure frequency. 

A comparison of the two groups regarding clinical factors revealed several statistically significant differences (Table 1). Univariate analysis showed that age at seizure onset, having mixed seizure types, history of status epilepticus, history of neonatal seizures, history of both having febrile and afebrile seizures, daily seizures at the onset, abnormality on the first electroencephalogram, generalized epileptic abnormality on electroencephalogram, abnormal neurodevelopmental status, abnormal neuroimaging, and having symptomatic etiology were significant risk factors for the development of drug-resistant epilepsy (p < 0.05).

**Table 1:** Clinical features of patients.

Clinical factors	Drug-resistantepilepsy (n =177)	Drug-responsive epilepsy (n = 281)	p-value
Age of onset	Mean: 1.85 years	Mean: 4.92years	<0.001
Mixed seizure type	28 (16%)	6 (2%)	<0.001
History of status epilepticus	50 (28%)	6 (2%)	<0.001
History of neonatal seizure	41 (23%)	23 (8%)	<0.001
History of both febrile and afebrile seizures	48 (27%)	32 (11%)	<0.001
Multiple seizures in a day	148 (83%)	54 (19%)	<0.001
Epileptiform discharge on initial EEG	172 (97%)	217 (77%)	<0.001
Generalized epileptic abnormality on EEG	59 (33%)	50 (18%)	<0.001
MRI abnormality	134 (76%)	108 (38%)	<0.001
Symptomatic etiology	153 (86%)	124 (44%)	<0.001
Intellectual disability	156 (88%)	102 (36%)	<0.001
Motor retardation	124 (70%)	72 (26%)	<0.001

Specific epileptic syndromes were detected in 43 patients (24%) in the drug-resistant group. These syndromes were Ohtaraha syndrome (n = 2), West syndrome (n = 28), Dravet syndrome (n = 7), Lennox-Gastaut syndrome (n = 2), Landau Kleffner Syndrome (n = 2), and progressive myoclonic epilepsy (Lafora disease in one patient and neuronal ceroid lipofuscinosis in one patient). 

In multivariable analysis, having mixed seizure types, history of status epilepticus, having multiple seizures in a day, intellectual disability, symptomatic etiology, and MRI abnormality remained significant predictors for developing drug-resistant epilepsy (Table 2). 

**Table 2:** Independent risk factors after logistic regression analysis.

	B	SE	OR	95% CI	p-value
Mixed seizure types	1.62	0.58	3.19	0.99–10.24	0.03
History of status epilepticus	1.92	0.53	5.09	1.71–15.09	0.01
Multiple seizures in a day	2.17	0.30	10.85	5.73–20.53	0.00
Intellectual disability	1.72	0.36	3.90	1.49–10.18	0.00
Symptomatic etiology	1.04	0.48	2.27	0.78–6.64	0.03
MRI abnormality	1.03	0.12	1.91	0.72–5.05	0.00

B: beta coefficient, SE: standard error, OR: odds ratio, CI: confidence interval.

## 4. Discussion

Several clinical features can be identified in the course of childhood epilepsy that may predict the outcome. Early prediction of drug-resistant epilepsy will guide determining the most appropriate treatment option for the patient. Several previous studies have reported factors that may predict drug-resistant epilepsy [5–8]. To the best of our knowledge, our study is the largest to be performed on a population of Turkish children with epilepsy. 

Having mixed seizure types was identified as a risk factor for drug-resistant epilepsy in most studies [5,7,8]. Previous studies have also stated that seizure types have an essential effect on prognosis, and children with multiple seizure types may have worse outcomes [5–8]. Similarly, in our research, mixed seizure types were an independent risk factor for refractoriness. 

In our study, the history of status epilepticus predicted drug resistance. Status epilepticus results from less inhibition and hyperexcitability, and as status epilepticus lasts longer, gamma-aminobutyric acid (GABA)-ergic function declines, and excitatory input continues, contributing to neuronal death [9]. However, some studies suggest that status epilepticus is not related to refractoriness [10]. The reason for conflicting results may be that status epilepticus can occur both as a result and a cause of drug-resistant epilepsy [7].

Like in our study, in most studies, initial seizure frequency was identified as a risk factor for drug resistance [7,8,10,11]. Repeated seizures have been shown to produce neuronal loss and mossy fiber sprouting in the hippocampus, which may cause the forming of recurrent excitatory circuits [12]. 

Patients with physical and intellectual disabilities were reported to have less probability of gaining seizure control than patients with idiopathic epilepsy [12]. A previous study from Turkey comparing 200 children with refractory epilepsy and 208 children with well-controlled epilepsy have stated mental and motor retardation as independent risk factors for drug-resistant epilepsy [8]. Similarly, in our study, intellectual disability was associated with refractoriness of seizures. 

In most previous studies, symptomatic etiology has been reported as a significant risk factor for drug-resistant epilepsy [1,8,11,13]. The changed structure of the central nervous system and function leads to hyperexcitability as the cause of epilepsy. Brain lesions result in neuronal death and reactive gliosis [14]. One of the mechanisms of drug-resistant epilepsy is the transporter hypothesis. According to this hypothesis, the structural abnormalities damage the capillary endothelial cells that constitute the blood-brain barrier, leading to the overexpression of efflux transports and drug resistance [14]. In our study, 86% of our cases with refractory seizures had symptomatic etiology, significantly higher than the drug-responsive group.

Epileptic patients with structural brain abnormalities have a lower chance of entering remission compared with structurally normal brains [12]. Previous studies revealed abnormal MRI findings as a predictor of refractoriness [8,15]. In our study, MRI abnormalities were significantly higher in children with drug-resistant epilepsy. 

In conclusion, this retrospective study highlights the factors predicting drug resistance in childhood epilepsy. Our study indicated strong univariate associations with several predictive factors. Moreover, our research points out that having mixed seizure types, history of status epilepticus, having multiple seizures in a day, intellectual disability, symptomatic etiology, and MRI abnormality are independent predictors of drug-resistant childhood epilepsy. One limitation of this study involves its methodology. This study is retrospective and should be verified with prospective studies. 

## Funding

The authors received no financial support for the research, authorship, and/or publication of this article.

## Informed consent and ethical approval

The study was approved by the Dokuz Eylül University Ethics Committee (report number 2013/42-08). Informed consent was obtained from the families of all participants before the study.
